# Individual risk and prognostic value prediction by machine learning for distant metastasis in pulmonary sarcomatoid carcinoma: a large cohort study based on the SEER database and the Chinese population

**DOI:** 10.3389/fonc.2023.1105224

**Published:** 2023-06-26

**Authors:** Xinglin Yi, Wenhao Xu, Guihua Tang, Lingye Zhang, Kaishan Wang, Hu Luo, Xiangdong Zhou

**Affiliations:** ^1^ Department of Respiratory Medicine, Southwest Hospital of Third Military Medical University, Chongqing, China; ^2^ Department of Urinary Medicine Center, Southwest Hospital of Third Military Medical University, Chongqing, China; ^3^ Department of Neurosurgery Department, Southwest Hospital of Third Military Medical University, Chongqing, China

**Keywords:** machine learning, SEER, Pulmonary sarcomatoid carcinoma, risk, prognosis

## Abstract

**Background:**

This study aimed to develop diagnostic and prognostic models for patients with pulmonary sarcomatoid carcinoma (PSC) and distant metastasis (DM).

**Methods:**

Patients from the Surveillance, Epidemiology, and End Results (SEER) database were divided into a training set and internal test set at a ratio of 7 to 3, while those from the Chinese hospital were assigned to the external test set, to develop the diagnostic model for DM. Univariate logistic regression was employed in the training set to screen for DM-related risk factors, which were included into six machine learning (ML) models. Furthermore, patients from the SEER database were randomly divided into a training set and validation set at a ratio of 7 to 3 to develop the prognostic model which predicts survival of patients PSC with DM. Univariate and multivariate Cox regression analyses have also been performed in the training set to identify independent factors, and a prognostic nomogram for cancer-specific survival (CSS) for PSC patients with DM.

**Results:**

For the diagnostic model for DM, 589 patients with PSC in the training set, 255 patients in the internal and 94 patients in the external test set were eventually enrolled. The extreme gradient boosting (XGB) algorithm performed best on the external test set with an area under the curve (AUC) of 0.821. For the prognostic model, 270 PSC patients with DM in the training and 117 patients in the test set were enrolled. The nomogram displayed precise accuracy with AUC of 0.803 for 3-month CSS and 0.869 for 6-month CSS in the test set.

**Conclusion:**

The ML model accurately identified individuals at high risk for DM who needed more careful follow-up, including appropriate preventative therapeutic strategies. The prognostic nomogram accurately predicted CSS in PSC patients with DM.

## 1 Introduction

Pulmonary sarcomatoid carcinoma (PSC) is rare among all lung malignancies, with an incidence of 0.1–0.5% (1, 2). According to the World Health Organization classification guidelines in 2021, PSC consists of giant cell carcinoma, pleomorphic carcinoma, spindle cell carcinoma, pulmonary blastoma, and carcinosarcoma (3). Clinically, newly diagnosed patients with PSCs are more likely to be male (77%), smokers (84%), and of advanced age, with a median age of 68 years at diagnosis (4). Similar to other subtypes of non-small cell lung carcinoma (NSCLC), the initial symptoms of PSC include cough, chest pain, weight loss, and dyspnea (5).

PSC is a highly aggressive malignancy. Recent studies have reported that the 5-year survival rate ranges from approximately 15% to 20% (6, 7), which is considerably lower than that in other subtypes of NSCLC. Targeted drugs, especially focusing on mesenchymal-epithelial transition (MET) exon 14 skipping mutations, have been shown to be beneficial in improving the median survival time to 10 months (8). However, distant metastasis (DM) still results in most patients having a shorter survival time (9–11). Owing to the rapid invasion into vasculature, 40–60% of PSC patients are diagnosed with DM at first presentation (6, 9, 12). The most common sites of DM are bone (16%), lungs (15%), brain (12%), and liver (8%), while 62% of patients present with multiple DM sites (13). The 1- and 3-year overall survival (OS) probabilities of PSC patients with DM are reported to be only 14.1% and 5.5%, respectively, which are significantly lower than those of patients without DM (58.2% and 38.1%, respectively) (13). Therefore, it is of great clinical significance to identify those at risk of developing DM upon initial stage of diagnosis.

Computed tomography (CT), magnetic resonance imaging (MRI), and positron emission tomography-computed tomography (PET-CT) are commonly used diagnostic modalities for screening for DM in newly hospitalized patients with PSC. However, these screening methods are difficult to apply to all hospitalized PSC patients due to their high cost, including time-related costs. Meanwhile, due to its rare incidence, the prognostic factors of PSC patients with DM remain unclear. Consequently, research investigating the factors influencing survival is important as it can inform and guide clinical strategies. Since the emergence of big data analysis and machine learning (ML), it is possible to provide an alternative option for factors screening. There have been several predictive models with outstanding performance being built to apply in clinical practice by using big data and ML (14–16). The Surveillance, Epidemiology, and End Results (SEER) database (https://seer.cancer.gov/) covers geographically diverse patients with detailed information on the patients’ clinicopathological statistics and follow-up visits, providing an abundance of medical cases to analyze. This real-world-based database offers a golden opportunity to develop or examine ML models in this field. However, to our knowledge, no studies have focused on establishing models for the prediction and prognosis of DM in PSC patients.

Therefore, this study aimed to establish and validate diagnostic and prognostic models based on ML algorithms and Cox regression through large population and ML. Additionally, this study aimed to offer personalized predictors that could serve as effective tools for clinicians to preliminarily evaluate the risk and prognosis of PSC with DM, saving patients from unnecessary costs.

## 2 Materials and methods

### 2.1 Data source and characteristics

Clinicopathological information of patients with PSC diagnosed between 2004 and 2018 was collected from the SEER database. Additionally, clinicopathological data from Southwest Hospital (2004–2022) in China was retrospectively obtained using an electronic medical record system. Inclusion criteria comprised the primary disease site and morphology, based on the 6th Edition of the American Joint Committee on Cancer (AJCC) “Lung,” with the following International Classification of Diseases for Oncology 3rd Edition (ICD-O-3) codes: 8022/3, 8031/3, 8032/3, 8072/3, or 8980/3. Exclusion criteria comprised patients whose information on laterality, primary site, tumor-node-metastasis (TNM) stage, histology, marital status, and tumor size was unknown; patients with more than one primary malignancy; patients aged <18 years; and patients whose survival time and therapeutic information were unknown. In addition, patients with unknown survival months were excluded from the survival analysis. Finally, a total of 844 patients from the SEER database and 94 patients from Southwest Hospital in China were included in the analysis. For the diagnostic model for DM, 844 patients in the SEER cohort were divided randomly into training and internal test sets in a 7:3 ratio with 589 and 255 cases, respectively. 94 patients from the Southwest Hospital cohort were assigned as the external validation set. For the prognostic model for cancer-specific survival (CSS) of PSC patients with DM, we randomly assigned PSC patients with DM into training and test groups in a 7:3 ratio. As a result, 270 PSC patients with DM were included in the training set, and 117 were included in the validation set. External examination for the prognostic model was not performed for two reasons: first, considerable prognostic information about the PSC patients with DM was censored in our hospital; second, the sample size was too small to satisfy the minimal sample size needed for analysis.

Clinicopathological factors included age, sex, histology, ethnicity, TNM stage, laterality, primary site, marital status, survival time, surgery, chemotherapy, and radiation therapy. Primary T and M staging was adjusted based on tumor size and extension according to the AJCC 8th Edition TNM staging system. Due to the sample limitations, ethnicity was categorized into ‘European’ and ‘other’ groups and marital status into ‘married’ and ‘other’ groups.

### 2.2 Statistical analysis

The overall statistical analysis was performed using software (version 4.2.1). Chi-square tests were used to discern differences in categorical variables, and t-tests were used to compare discrepancies between the continuous variables. All variables were included in the correlation analysis using the Spearman method, which was performed to determine which variables were significant and to exclude confounding variables.

### 2.3 Development and evaluation of ML-based diagnostic models for DM

In the preliminary analysis, variables with P < 0.05 in the univariate logistic analysis in the training set were included in the model construction process, which involved the logistic regression (LR), random forest (RF), support vector machine (SVM), extreme gradient boosting (XGB), decision tree (DT), and artificial neural network (ANN) models. The hyperparameters were tuned using 10-fold cross-validation and a grid search procedure to improve the predictive performance and enhance the stability of the ML models. The “tidymodels” package in R software completed all these development procedures.

Model performance was comprehensively evaluated using the area under the curve (AUC), sensitivity, specificity, and accuracy. In addition, we performed calibration curve and decision curve analysis (DCA) to check the discriminative ability and practical clinical value. The best-performing model was then used to build a web-based calculator to allow access to the model.

### 2.4 Establishment and validation of the prognostic nomogram

In the training cohort, a univariate Cox model was used to identify CSS-related independent factors for PSC patients with DM. Variables with P < 0.05 were included in a multivariate Cox analysis. The variables with P < 0.05 in the multivariate Cox regression were incorporated into the prognostic nomogram development to estimate survival probability at 3 and 6 months. The AUC, calibration, and DCA estimators were used to evaluate the nomogram.

## 3 Results

### 3.1 Baseline characteristics and correlation analysis

**Figure 1:**
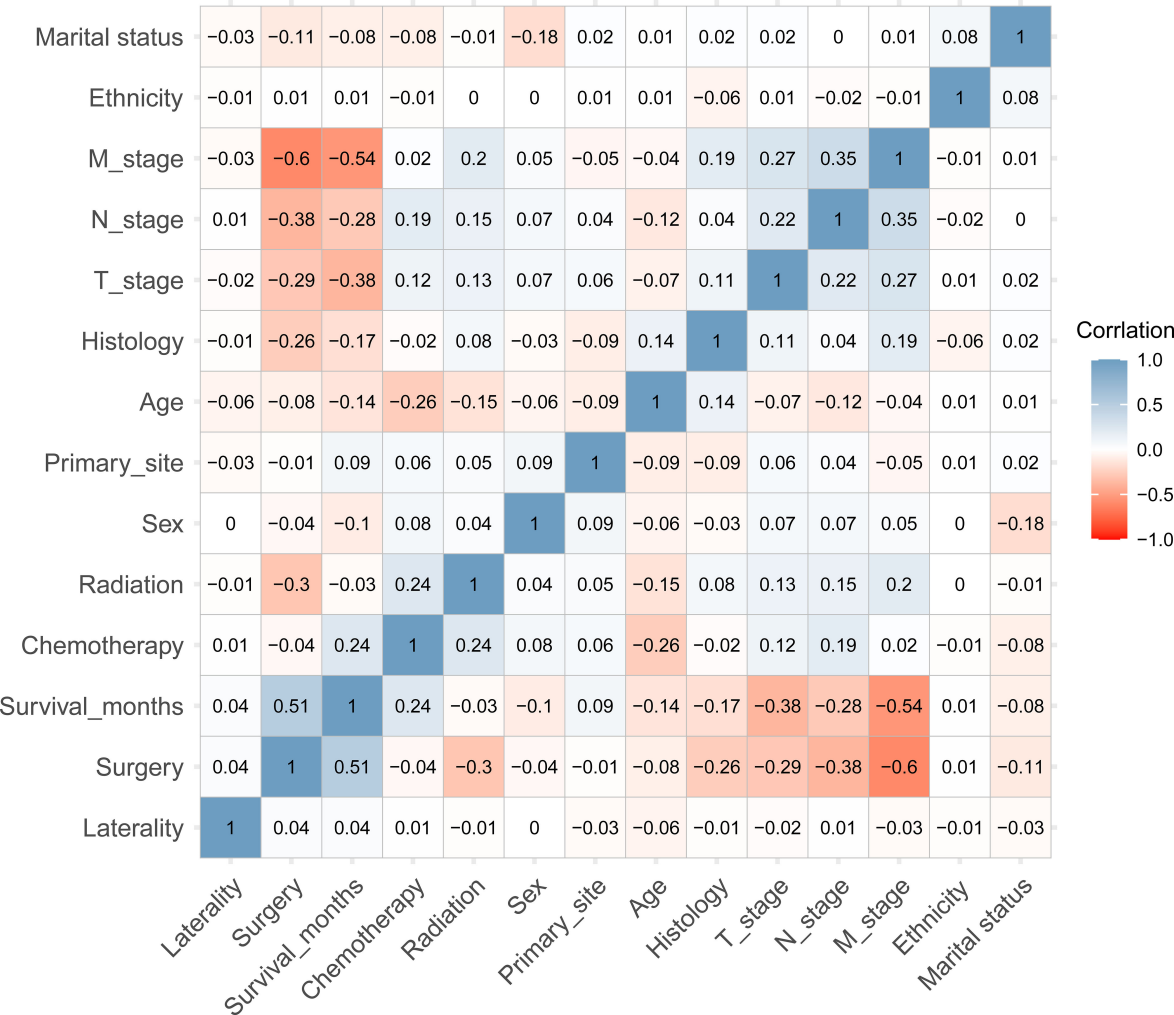
The heatmap of Spearman’s correlation analysis of the baseline characteristics. The correlation index ranges from -1.0 to 1.0, with a brighter color indicating a stronger correlation.

A total of 589 patients with PSC were included into the training set while 255 PSC patients were included into in the internal test set. 270 patients in the training set and 117 patients in the internal test set were accompanied with DM. In addition, 94 patients whose clinical information was recorded from the Southwest Hospital were assigned to an external test set, among whom 37 patients suffered from DM. The detailed characteristics and discrepancies between the DM and non-DM groups are presented in **Table 1**. Patients with DM were observed to be more likely male with advanced T and N stages. In addition, the histology of codes 8031 and 8032, namely giant cell carcinoma and spindle cell carcinoma, respectively, were found to be correlated with a higher proportion of DM. Spearman’s correlation analysis suggested that T-stage, N-stage, radiation therapy, and histology were positively correlated with DM, whereas surgery and survival months were negatively correlated. In terms of survival, surgery and chemotherapy were observed to positively influence survival time. In contrast, older age, T stage, N stage, and M stage negatively contributed to survival (**Figure 1**).

**Table 1:** Baseline characteristics.

Variables	Train set		Internal test set			External test set	
	Non-DM (N = 319)	DM (N = 270)	Non-DM (N = 138)	DM (N = 117)		Non-DM (N = 57)	DM (N = 37)
T stage							
T1	45 (14.1%)	14 (5.2%)	30 (21.7%)	8 (6.8%)		16 (28.1%)	3 (8.1%)
T2	95 (29.8%)	45 (16.7%)	37 (26.8%)	22 (18.8%)		22 (38.6%)	14 (37.8%)
T3	86 (27.0%)	69 (25.6%)	31 (22.5%)	26 (22.2%)		9 (15.8%)	11 (29.7%)
T4	93 (29.2%)	142 (52.6%)	40 (29.0%)	61 (52.1%)		10 (17.5%)	9 (24.3%)
N stage							
N0	202 (63.3%)	86 (31.9%)	83 (60.1%)	41 (35.0%)		40 (70.2%)	9 (24.3%)
N1	42 (13.2%)	31 (11.5%)	22 (15.9%)	14 (12.0%)		7 (12.3%)	1 (2.7%)
N2	67 (21.0%)	109 (40.4%)	31 (22.5%)	43 (36.8%)		10 (17.5%)	14 (37.8%)
N3	8 (2.5%)	44 (16.3%)	2 (1.4%)	19 (16.2%)		0 (0%)	13 (35.1%)
Histology							
8022	155 (48.6%)	75 (27.8%)	81 (58.7%)	25 (21.4%)		14 (24.6%)	4 (10.8%)
8031	57 (17.9%)	96 (35.6%)	24 (17.4%)	39 (33.3%)		9 (15.8%)	9 (24.3%)
8032	90 (28.2%)	95 (35.2%)	29 (21.0%)	50 (42.7%)		20 (35.1%)	15 (40.5%)
8972	1 (0.3%)	0 (0%)	1 (0.7%)	0 (0%)		5 (8.8%)	4 (10.8%)
8980	16 (5.0%)	4 (1.5%)	3 (2.2%)	3 (2.6%)		9 (15.8%)	5 (13.5%)
Sex							
Female	135 (42.3%)	101 (37.4%)	60 (43.5%)	44 (37.6%)		9 (15.8%)	9 (24.3%)
Male	184 (57.7%)	169 (62.6%)	78 (56.5%)	73 (62.4%)		48 (84.2%)	28 (75.7%)
Laterality							
Center	136 (42.6%)	116 (43.0%)	54 (39.1%)	57 (48.7%)		31 (54.4%)	14 (37.8%)
Right	183 (57.4%)	154 (57.0%)	84 (60.9%)	60 (51.3%)		26 (45.6%)	23 (62.2%)
Primary site							
Lower	95 (29.8%)	81 (30.0%)	29 (21.0%)	40 (34.2%)		18 (31.6%)	14 (37.8%)
Middle	14 (4.4%)	18 (6.7%)	10 (7.2%)	5 (4.3%)		1 (1.8%)	1 (2.7%)
Other	20 (6.3%)	15 (5.6%)	4 (2.9%)	7 (6.0%)		/	/
Upper	190 (59.6%)	156 (57.8%)	95 (68.8%)	65 (55.6%)		38 (66.7%)	22 (59.5%)
Ethnicity							
Other	23 (7.2%)	23 (8.5%)	12 (8.7%)	9 (7.7%)		57 (100%)	37 (100%)
European	296 (92.8%)	247 (91.5%)	126 (91.3%)	108 (92.3%)		/	/
Age							
Mean (SD)	67.4 (11.5)	67.4 (11.4)	66.7 (10.6)	64.5 (12.0)		63.2 (11.8)	65.4 (11.0)
Marital status							
Married	174 (54.5%)	138 (51.1%)	71 (51.4%)	64 (54.7%)		56 (98.2%)	37 (100%)
Other	145 (45.5%)	132 (48.9%)	67 (48.6%)	53 (45.3%)		1 (1.8%)	0 (0%)

DM, distant metastasis; SD, standard deviation.

### 3.2 Establishment and performance of diagnostic ML models for DM

**Figure 2:**
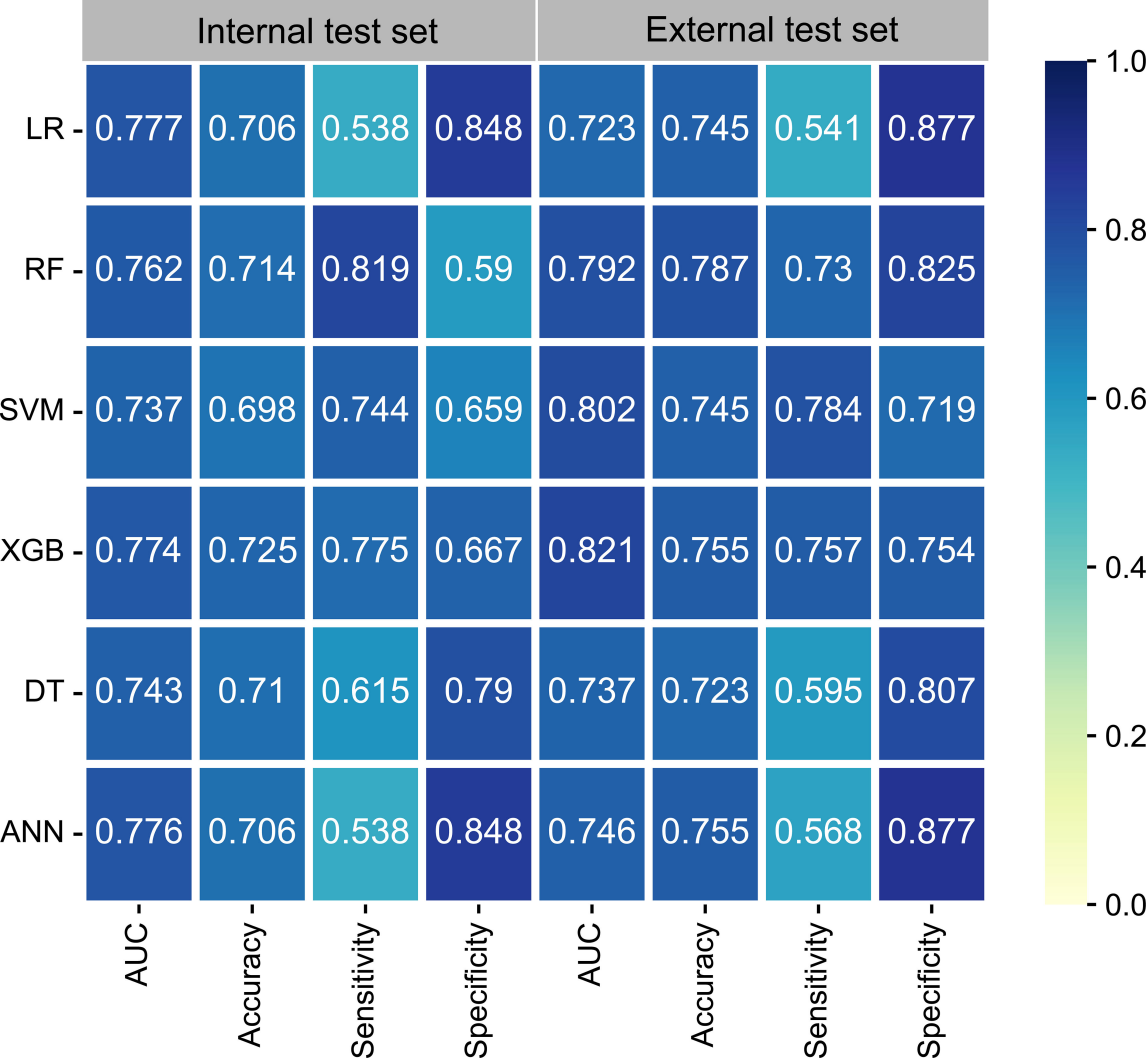
The performance of six ML models in terms of AUC, accuracy, sensitivity, and specificity. ANN, artificial neural network; AUC, area under the curve; DT, decision tree; LR, logistic regression; ML, machine learning; RF, random forest; SVM, support vector machine; XGB, extreme gradient boosting.

The univariate logistic regression analysis (**Supplementary Figure 1**), showed that T-stage, N-stage, and histology were variables with P-values < 0.05 and were therefore included in the ML models. In addition, the multivariate analysis (**Supplementary Figure 2**) showed that the N3 stage, T4 stage, N2 stage, histology of 8031, T3 stage, and histology of 8032 (arranged from high to low according to odds ratios [ORs]) were identified as significant factors contributing to DM. Six ML learning algorithms were established by incorporating the above selection of variables, comprising logistic regression (LR), extreme gradient boosting (XGB), random forest (RF), support vector machine (SVM), decision tree (DT), and artificial neural network (ANN) algorithms. Hyperparameters were fine-tuned by performing 10-cross validation and grid searches. Finally, LR, ANN, and XGB were found to have the highest AUC in the internal test set (**Figure 2**) while the XGB algorithm outperformed the others with the highest AUC of 0.821 in the external test set. The best hyperparameter metric was eta, 0.007; max_depth, 1L; gamma, 0.011; colsample_bytree, 1; colsample_bynode, 0.231; min_child_weight, 8L; and subsample, 0.997.

**Figure 3:**
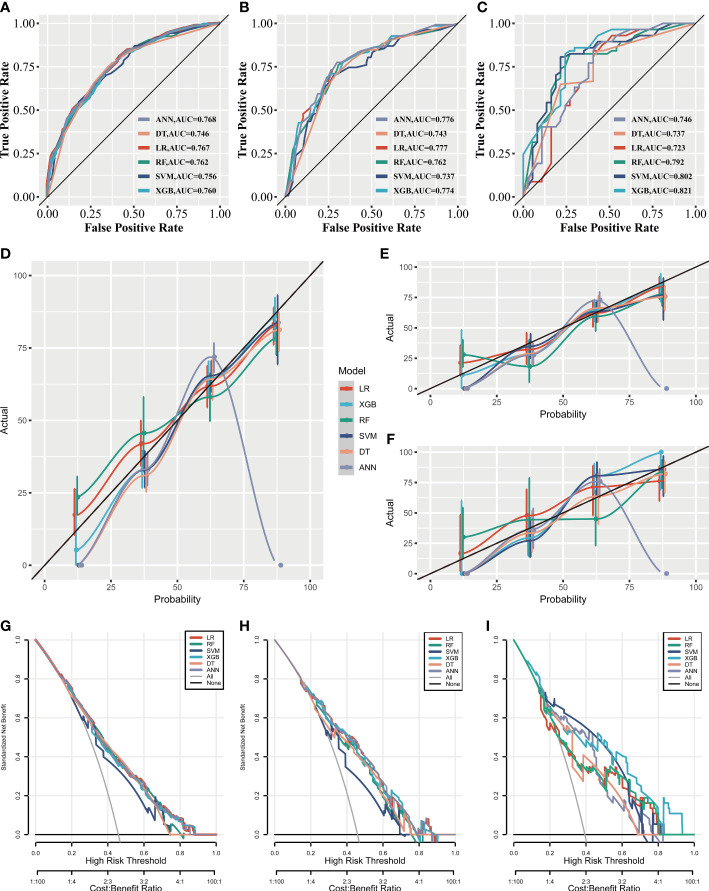
The clinical applicative performance of six ML models. Receiver operating characteristic curves of six ML models in the training set **(A)**, the internal test set **(B)**, and the external test set **(C)**. Calibration curves of six ML models in the training set **(D)**, the internal test set **(E)** and the external test set **(F)**. Decision curve analysis of six ML models in the training set **(G)**, the internal test set **(H)**, and the external test set **(I)**.

As illustrated in **Figures 3A–C**, the AUC differed slightly in the training set and the internal test set among the six ML algorithms; however, in the external test set, XGB performed the best. Calibration plots (**Figures 3D–F**) showed that ML algorithms had a good fitting ability, except for the ANN model, which meant that the ML algorithms accurately predicted the outcome. DCA curves (**Figures 3G–I**) suggested that ML algorithms had a high clinical application value and could effectively help diagnose DM.

### 3.3 Web-based predicator publication

**Figure 4:**
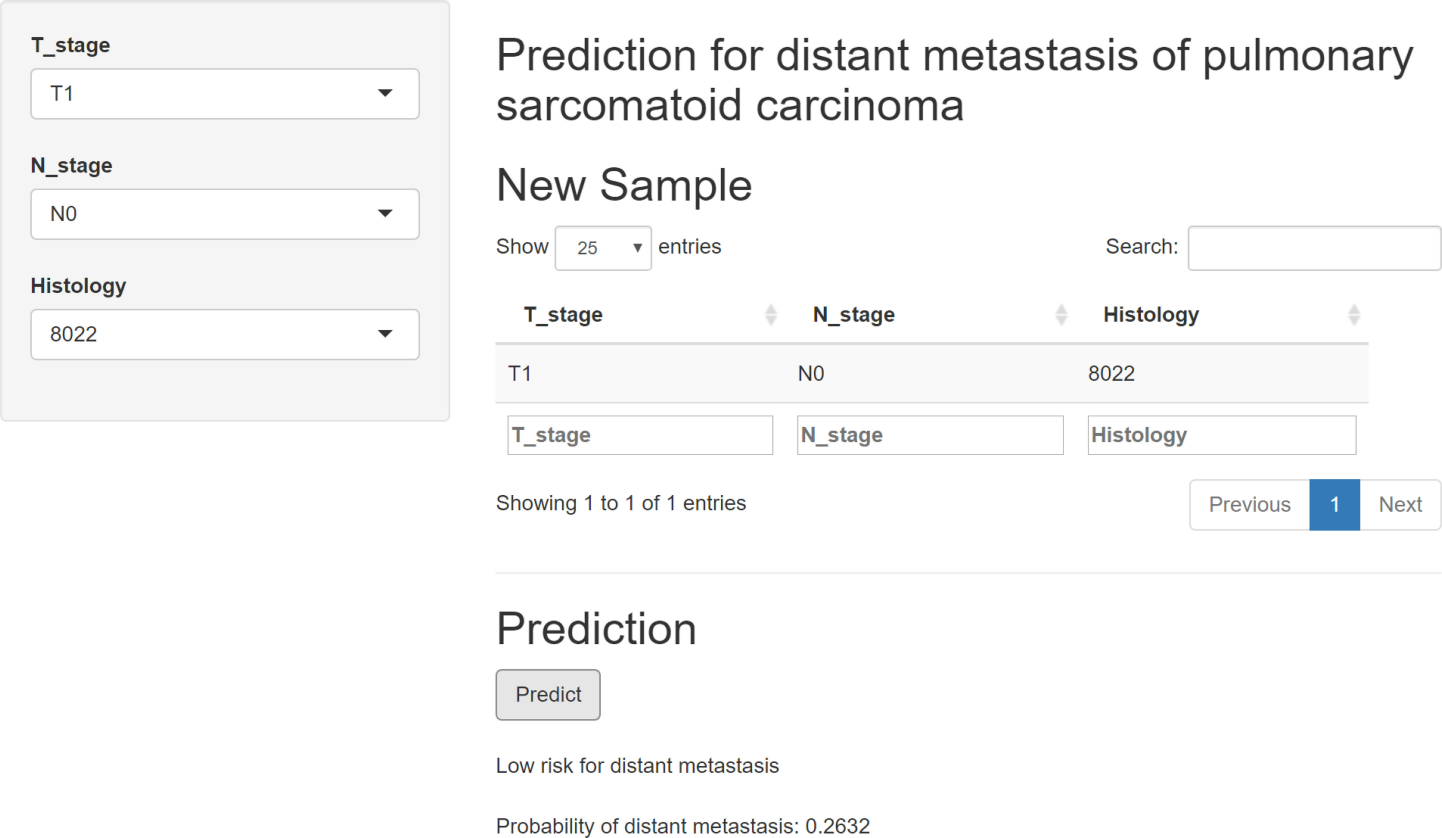
Web-based calculator online for free, based on the XGB model. XGB, extreme gradient boosting.

An online calculator based on the XGB model was successfully built (**Figure 4**), which performed the best among the other models. This web-based tool can be readily accessed at https://onepiece.shinyapps.io/PSCdistant/. This tool encompassed three simple clinical variables and could help clinicians accurately and conveniently identify those at risk for DM among patients with PSC.

### 3.4 Prognostic nomogram establishment and performance

**Figure 5:**
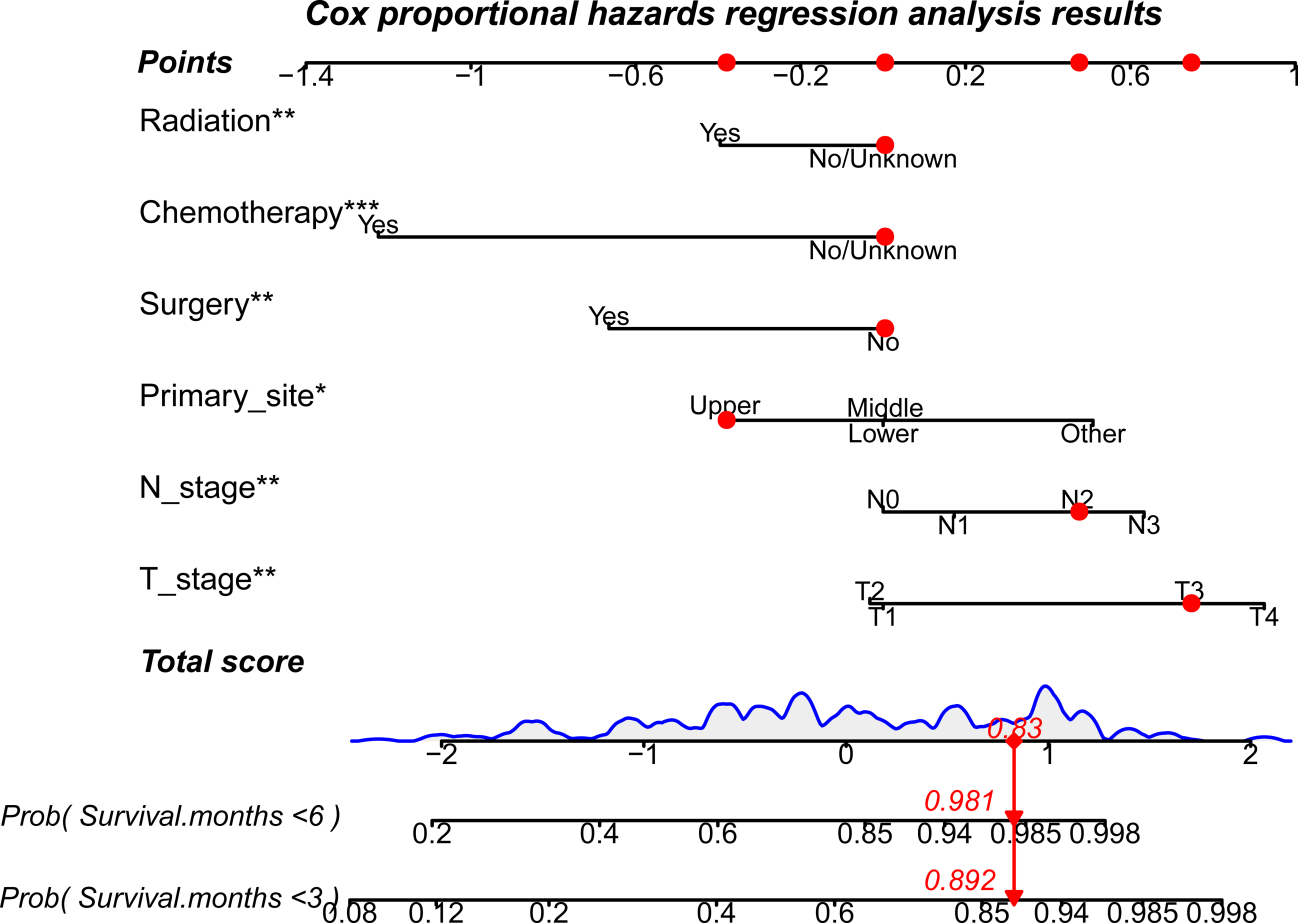
A prognostic nomogram to evaluate 3- and 6-month CSS-related survival probabilities for PSC patients with DM. Note: Drawing a vertical line from each line of the parameters to the “points” axis can be used to acquire the points for each variable. Then, the total score can be obtained by adding all the points for each independent variable. Finally, by drawing a vertical line from the “total score” axis to the survival probability line, the individual survival probability at 3 and 6 months can be calculated, and the risk level can be obtained. DM, distant metastases; PSC, pulmonary sarcomatoid carcinoma. *P<0.05; **P<0.01; ***P<0.001.

**Figure 6:**
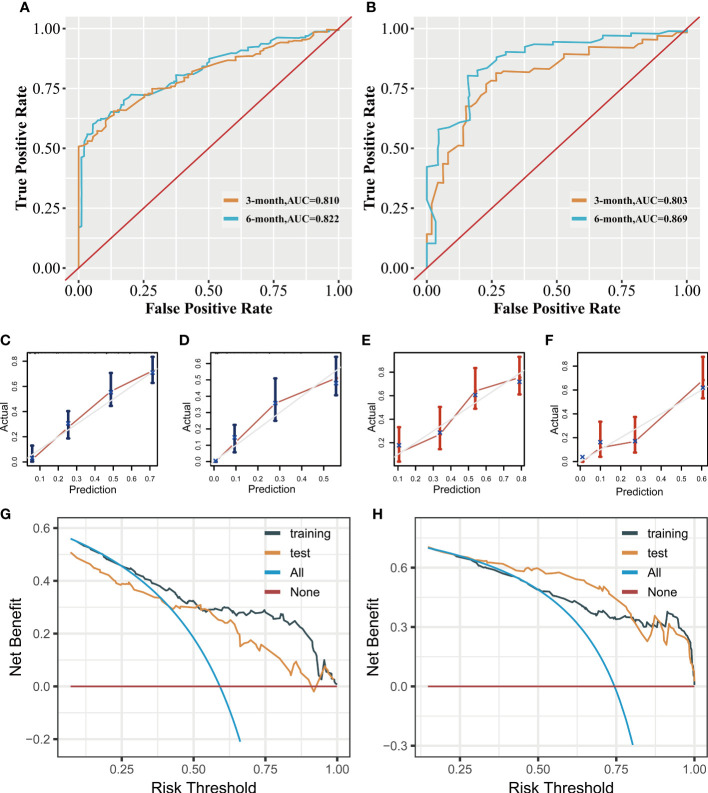
Clinical applicative performance of the prognostic nomogram. Receiver operating characteristic curves at 3 and 6 months in training **(A)** and test sets **(B)**. Calibration plots at 3 months **(C)** and 6 months **(D)** in the training set. Calibration plots at 3 months **(E)** and 6 months **(F)** in the test set. Decision clinical analysis at 3 and 6 months in the training **(G)** and test sets **(H)**.

In the prognostic analysis, 270 PSC patients with DM in the training and 117 patients in the test set were enrolled. As shown in **Supplementary Figure 3**, the univariate Cox regression analysis indicated that the T4 stage (P = 0.013), N2 stage (P = 0.048), N3 stage (P = 0.001), upper site (P = 0.023), surgery (P = 0.013), chemotherapy (P < 0.001), and radiation therapy (P < 0.001) were significantly associated with prognosis in PSC patients with DM. After performing multivariate Cox analysis (**Supplementary Figure 4**), stage T4 (P = 0.005), T3 (P = 0.034), N3 (P = 0.002), N2 (P = 0.005), and upper site (P = 0.01) were found to be independent adverse prognostic factors, whereas radiation therapy (P = 0.004), surgery (P = 0.004), and chemotherapy (P < 0.001) were protective prognostic factors for PSC patients with DM. We developed a prognostic nomogram based on these independent variables, to predict the CSS-related survival probability at 3 and 6 months (**Figure 5**). The CSS-related AUC values at 3 and 6 months were 0.810 and 0.822 in the training set (**Figure 6A**) and 0.803 and 0.869 in the test set (**Figure 6B**), respectively, suggesting high predictive accuracy. In addition, calibration (**Figures 6C–F**) and DCA curves (**Figures 6G, H**) showed the prognostic nomogram’s good fitting ability and clinical application.

## 4 Discussion

PSC presents a rare lung malignancy with a high probability for DM. Several studies having reported that DM is an independent risk factor for PSC (6, 17, 18). In this study, approximately 45.2% patients had DM at initial presentation, similar to that reported by Zombori-Tóth and Terra (19, 20). The high rate of DM may be attributed to its capacity to invade into vasculature and the high likelihood of DM recurrence. Lococo et al. analyzed the pathological results of 143 patients and found the high incidences of lymphatic (30%) and vascular emboli (68%) (21). Liang et al. found that MET mutations were commonly expressed in PSCs with a high rate of 16% (22), whereas the incidence of KRAS mutations was 22%. Similarly, Liu et al. have identified that eight out of 36 (22%) patients harboring MET mutations (23). This incidence was considerably more frequent than that in other NSCLC subtypes (3%) (24). A study on 77 Chinese patients with PSC indicated that positive METex14 skipping mutations rate was 20.8%, leading to worse disease-free survival (DFS) (25). In recent years, immune checkpoint inhibitors (ICIs) and targeted drugs for MET exon 14 skipping mutations have exhibited satisfying benefits in improving patient survival (6, 26, 27). However, patients who developed DM still had a significantly worse prognosis. Thus, early attention to DM is important to improve prognosis and help clinicians make the most optional targeted therapeutic decision.

CT, MRI, and PET-CT are conventional radiological screening methods for patients with NSCLC. However, performing all these radiological examinations is costly for newly diagnosed patients. In addition, time-consuming screening processes and potential side effects can delay the patients’ course from diagnosis to therapy. Therefore, there is an urgent need for a noninvasive, precise, and rapid method to assist in identifying potential DM at the initial hospitalization stage and to estimate the prognosis for PSC patients with DM. In this study, six ML algorithms for predicting DM in patients with PSC and a nomogram for evaluating the prognosis for those with DM were developed. An automatic calculator based on the best-performing algorithm was created and published online for public access. Moreover, the prognostic nomogram could accurately identify risk factors in PSC patients with DM and help clinicians evaluate survival.

Few studies have identified the risk factors for DM in patients with PSC due to its rarity. Logistic regression analysis was performed and found that histologic subtype, T stage, and N stage were independent risk factors for DM in patients with PSC. Among these factors, the N3 stage was most correlated with DM. Patients with the N3 stage had the highest OR of 9.72 (P < 0.001), followed by the T4 stage (OR 4.30, P < 0.001), N2 stage (OR 3.09, P < 0.001), giant cell carcinoma (OR 2.56, P < 0.001), T3 stage (OR 2.36, P = 0.022), and spindle cell carcinoma (OR 2.12, P < 0.001). These findings were similar to those reported by Xiao et al., who retrospectively analyzed 934 PSC patients using the SEER program database and reported that spindle cell carcinoma (OR 3.151, P < 0.001) and giant cell carcinoma (OR 4.023, P < 0.001) were independent risk factors for DM (13).

Similarly, T-stage and N-stage have been reported as risk factors for DM in lung adenocarcinoma and squamous cell carcinoma (28, 29). The two variables have also been identified as important factors for the development of DM in patients with PSC (28, 29). These three clinical features were then incorporated into the building procedures of the six ML models. The XGB model performed better than the other algorithms, with an AUC of 0.821, showing excellent predictive ability for DM in patients with PSC. In addition, calibration and DCA curves indicated that the model was highly consistent with actual observations and showed better clinical applicability (30).

Carcinoembryonic antigen levels, cytokeratin 19 fragment (CYFRA21-1), and other serologic indicators which were associated with DM in patients with NSCLC (31, 32) have not been included in this study due to missing data in the SEER program. However, the relatively high accuracy of the XGB algorithm still helped identify those PSC patients with a high risk for DM. To facilitate the use of the ML predictor, a web-based calculator was developed, which is likely to help clinicians detect DM early in patients with PSC in a convenient manner.

Compared to other subtypes of NSCLC, PSC is less sensitive to chemoradiotherapy. However, studies have confirmed that chemoradiotherapy significantly prolonged the CSS of PSC patients with DM. Xiao et al. analyzed the prognostic risk factors of 512 patients with metastatic PSC diagnosed between 1975 and 2016 in the SEER program (13). They found that having received chemotherapy (HR 0.308, P < 0.001) was an independent prognostic risk factor for PSC patients with DM, which was similar to our study findings (HR 0.29, P < 0.001). However, they did not identify radiation therapy and surgical resection as independent prognostic factors for PSC patients with DM, while these two therapies were indicated to prolong CSS significantly in our study. We speculate that this discrepancy was due to diagnosis time of the enrolled patients were prior to the year 2000 while surgical and radiation techniques were not so effective. Our study identified T-stage and N-stage as important prognostic risk factors for PSC patients with DM. Advanced T stage (T4, HR 2.52, P = 0.005; T3, HR 2.10, P = 0.03) and N-stage (N3, HR 1.88, P = 0.002; N2, HR 1.60, P = 0.005) correlated with poor CSS in PSC patients suffered from DM.

Interestingly, we have also found that the nodule site appeared to affect the prognosis for PSC patients with DM. CSS was longer in patients whose primary site was located on an upper rather than a lower lobe (HR, 0.68; P = 0.011). The reason may be that the nodules on the lower lung will develop more DM lesions leading a worse prognosis (13). Notably, we established a nomogram for predicting the prognosis for PSC patients with DM, with a relative higher AUC at 3 months and 6 months respectively. Its high consistency to actual observations indicates that this nomogram could precisely predict CSS in PSC patients with DM.

There are some limitations in our present study. First, this was a retrospective study, and selective bias could not be avoided. Second, characteristic information about DM was collected during initial hospitalization, which may have led to an underestimated percentage of DM in patients with PSC. Lastly, the predictive model was externally validated using patients’ information from Southwest Hospital in China. However, since this hospital mainly treats Chinese patients, the model’s application value should be further validated in cohorts involving differing ethnicities.

## 5 Conclusion

Our study indicated that PSC patients with advanced T-stage, N-stage, histology of giant cell carcinoma and spindle cell carcinoma were risk factors for DM and should receive more attention in terms of preventative therapeutic strategies. The AUC, accuracy, sensitivity, and specificity of the XGB model reached 0.821, 0.755, 0.757, and 0.754, respectively. A diagnostic model for DM based on the ML algorithm and a web-based predictor was then established, which could conveniently and precisely predict the risk of DM in PSC patients. Our study provided initial predictive and prognostic models for PSC patients with DM. Future studies may focus on further improving the models by adding other potential variables and developing more detailed models to predict the risk and prognosis for specific metastatic sites.

## Data availability statement

The datasets presented in this study can be found in online repositories. The names of the repository/repositories and accession number(s) can be found in the article/**Supplementary Material**.

## Ethics statement

The studies involving human participants were reviewed and approved by Southwest Hospital, Third Military Medicine University. Written informed consent for participation was not required for this study in accordance with the national legislation and the institutional requirements.

## Author contributions

XY and WX designed the study. GT, LZ, and KW collected the primary data. XY drafted the manuscript. HL and XZ reviewed and edited the paper. All authors contributed to the article and approved the submitted version.

## Supplementary material

Supplementary Figure 1 The Forest plot of the univariate logistic regression analysis. OR, odds ratio.

Supplementary Figure 2 Forest plot of multivariate logistic regression analysis. The OR value decreases from top to bottom.

Supplementary Figure 3 The Forest plot of the univariate Cox proportional hazards regression analysis. HR, hazard ratio.

Supplementary Figure 4 The Forest plot of the multivariate Cox proportional hazards regression analysis. The HR value is in descending order from top to bottom.
