# Supplementary figures and images for: Individual risk and prognostic value prediction by machine learning for distant metastasis in pulmonary sarcomatoid carcinoma: a large cohort study based on the SEER database and the Chinese population
Source: Front Oncol. 2023 Jun 26;13:1105224. doi: 10.3389/fonc.2023.1105224 (PMC10332636; doi:10.3389/fonc.2023.1105224)

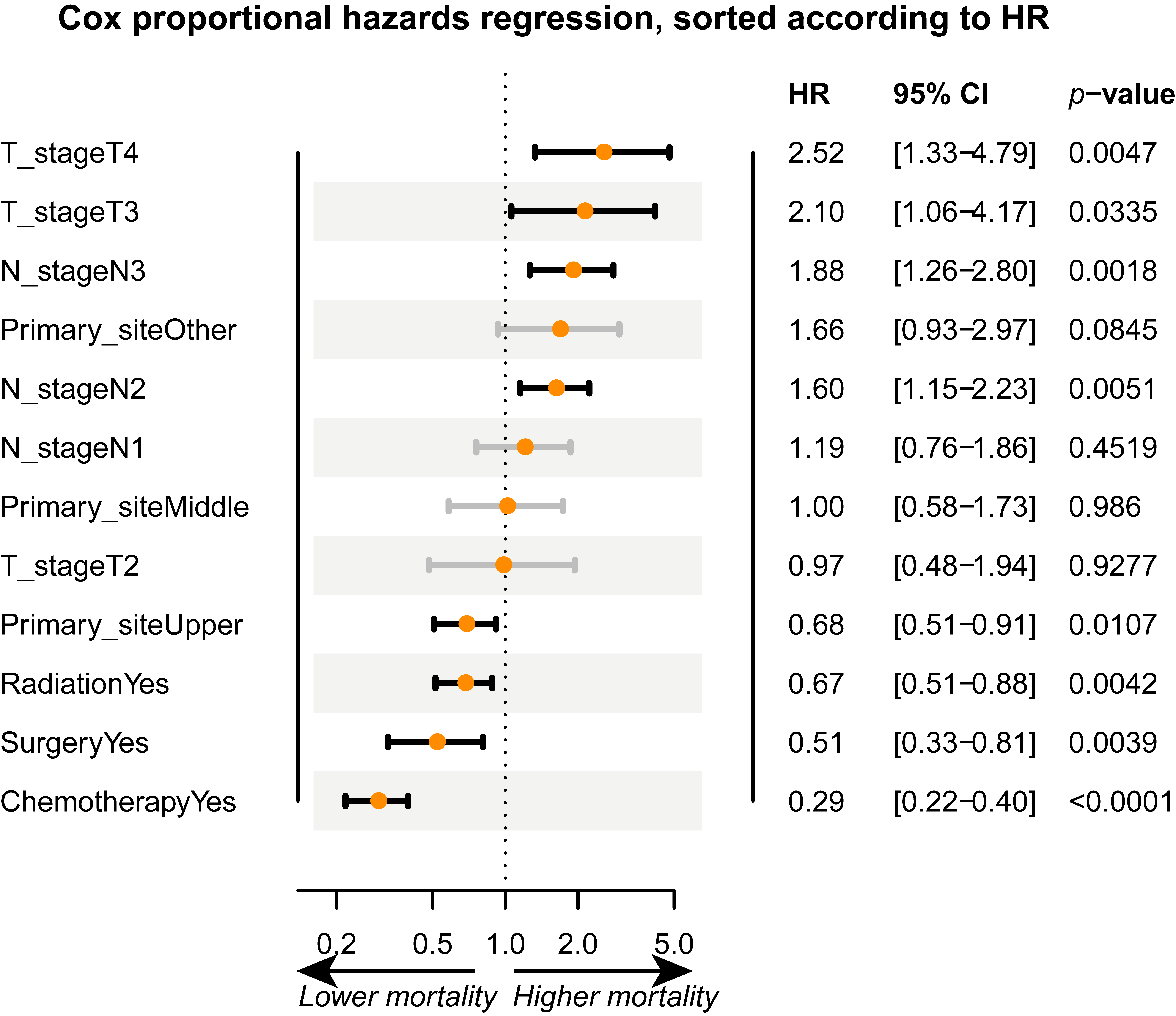

Supplement: Supplementary Figure 4 — The Forest plot of the multivariate Cox proportional hazards regression analysis. The HR value is in descending order from top to bottom. [file Image_4.tif]
